# Antireflux Surgery for Barrett's Esophagus: Where Do We Stand in Preventing Esophageal Adenocarcinoma?

**DOI:** 10.1111/nyas.70196

**Published:** 2026-02-06

**Authors:** Dagmar Kollmann, Cansel Etyemez, Reza Asari, Gerd Jomrich, George Triadafilopoulos, Kenneth J. Vega, Bruno Zilberstein, Margaret J. Zhou, Qin Huang, Hiroshi Mashimo, Eun Ji Shin, John O. Clark, Sebastian F. Schoppmann

**Affiliations:** ^1^ Department of General Surgery Medical University of Vienna Vienna Austria; ^2^ UT MD Anderson Cancer Center Houston Texas USA; ^3^ Gastroenterology and Hepatology Augusta University Medical College of Georgia Augusta Georgia USA; ^4^ Sao Leopoldo Mandic School of Medicine Campinas Brazil; ^5^ Division of Gastroenterology and Hepatology, Department of Medicine Stanford University School of Medicine Redwood City California USA; ^6^ Department of Pathology Beth Israel Deaconess Medical Center Boston Massachusetts USA; ^7^ Department of Medicine, VA Boston Healthcare System Harvard Medical School Boston Massachusetts USA; ^8^ Division of Gastroenterology and Hepatology, Department of Medicine Johns Hopkins Medical Institutions Baltimore, Mayland USA

**Keywords:** antireflux surgery, Barrett's esophagus, esophageal adenocarcinoma

## Abstract

Esophageal adenocarcinoma is a major global health concern, primarily arising from gastroesophageal reflux disease, with Barrett's esophagus being its main precursor. Although proton pump inhibitors are commonly used to manage the symptoms from gastroesophageal reflux disease, their role in preventing esophageal adenocarcinoma progression remains uncertain. The aim of this review is to summarize the current advances in the diagnosis of Barrett's esophagus and its progression, as well as to critically evaluate and compare the impact of antireflux surgery on Barrett's esophagus and its potential role in preventing its progression to esophageal adenocarcinoma. In conclusion, surgical intervention, particularly antireflux surgery, has been associated with reduced esophageal adenocarcinoma risk in some studies, offering better long‐term symptom control and possibly preventing cancer progression. However, other authors suggest that the cancer risk does not decrease significantly with surgery, highlighting the need for further investigation into its long‐term preventive benefits. Several novel strategies have been established over the last few years that will facilitate an early diagnosis of Barrett's esophagus in the future.

## Graphical abstract

Esophageal adenocarcinoma (EAC) often develops from gastroesophageal reflux disease (GERD), with Barrett's esophagus (BE) as its main precursor. While proton pump inhibitors manage GERD symptoms, their preventive role in EAC remains unclear. This review summarizes advances in diagnosing BE and evaluates anti‐reflux surgery for its potential to prevent progression. Although evidence is mixed, emerging technologies and personalized approaches are shaping a new standard in BE prevention and treatment.

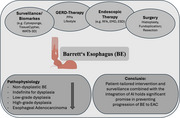

## 1 Introduction

Esophageal cancer is the 11th most commonly diagnosed cancer and the seventh leading cause of cancer death worldwide, with an estimated 511,000 new cases and 445,000 deaths in 2022 [1]. While esophageal squamous cell carcinoma (ESCC) incidence has declined in many areas, the incidence of esophageal adenocarcinoma (EAC) has risen sharply in Europe, North America, and Australia over the past four decades—a trend that is expected to continue [2]. Until 2030, the number of new EAC cases is projected to increase significantly across all studied countries due to rising risk factors and demographic changes, whereas ESCC incidence is expected to decline further. By 2030, 1 in 100 men in the Netherlands and the United Kingdom is predicted to develop EAC in their lifetime [3].

The development of EAC is primarily linked to chronic gastroesophageal reflux disease (GERD). Persistent acid exposure damages the normal squamous epithelium of the esophagus, leading to its transformation into a specialized columnar epithelium, known as Barrett's esophagus (BE), through mostly unknown pathways. This metaplastic change increases the risk of further progression, as BE can develop into low‐grade dysplasia (LGD), then high‐grade dysplasia (HGD), and eventually invasive esophageal EAC [4]. The inflammatory environment at the esophagogastric junction (EGJ) plays a major role in whether BE progresses to EAC, where several factors might be triggered by cytokines and other regulatory signaling mechanisms [1].

## 2 Methods

### 2.1 Search Strategy

A comprehensive literature search was conducted using PubMed, Google Scholar, and the Cochrane Library to identify relevant studies on the role of surgical and medical interventions in preventing EAC in patients with BE. The search included studies published in English without time restrictions to ensure a broad inclusion of relevant data.

The primary search terms used were “esophageal adenocarcinoma,” “Barrett's esophagus,” “antireflux surgery,” and “antireflux medication,” among others. Additional keywords and Medical Subject Headings (MeSH) terms were applied where appropriate to enhance search sensitivity. Reference lists of key articles were also screened to identify additional relevant studies.

The studies included in Table 1 were selected based on their relevance to the prevention of EAC through antireflux interventions, including both surgical and pharmacological approaches. Only peer‐reviewed studies, systematic reviews, meta‐analyses, randomized controlled trials (RCTs), and large cohort studies were considered.

**TABLE 1:** Summary of studies focused on the effect of antireflux surgery on the development and progress of Barrett's esophagus.

Reference	Year	Type of study	Number of patients	Treatment (patients)	Major outcomes
Parrilla et al. [69]	2003	Randomized	101	Medical (43) Surgical (58)	Both approaches achieved similar clinical success rates of 91% in symptom control. However, ARS was more effective in controlling acid reflux and preventing progression to dysplasia or adenocarcinoma, particularly when surgical intervention was successful.
Chang et al. [70]	2007	Systematic review	1696	Medical (996) Surgical (700)	Surgery was associated with a higher probability of regression of BE and/or dysplasia compared to medical therapy. However, the evidence suggesting that surgery reduces the incidence of EAC is largely driven by uncontrolled studies, indicating a need for further research.
Maret‐Ouda et al. [71]	2016	Systematic review and meta‐analysis	10,243	Medical (4596) Surgical (5647)	ARS was associated with a reduced risk of EAC compared to medical therapy in patients with BE, with a pooled incidence rate ratio (IRR) of 0.46 (95% CI 0.20–1.08). However, the EAC risk after ARS did not revert to that of the background population.
Markar et al. [72].	2018	National population‐based cohort	19,896	Surgical	ARS was associated with a 36% reduction in esophageal cancer risk among patients with GERD (HR = 0.64; 95% CI 0.52–0.78). However, in patients with BE, the reduction was not statistically significant (HR = 0.47; 95% CI 0.12–1.90).
Maret‐Ouda et al. [73]	2021	Multicenter, population‐based cohort	38,250	Surgical	The long‐term risk of EAC after ARS in patients with GERD was investigated. The study found that EAC risk did not decrease after surgery, among those with severe GERD, and/or BE.
Åkerström et al. [74]	2024	Prospective cohort	3506	Medical (2086) Surgical (1420)	Patients who undergo ARS (1420) because of GERD do not have a lower risk of EAC than those using antireflux medication (2086). Instead, patients with BE who undergo ARS remain at an increased risk of EAC and should continue taking part in surveillance programs.

Abbreviations: ARS, antireflux surgery; BE, Barret's esophagus; CI, confidence interval; EAC, esophageal adenocarcinoma; GERD, gastroesophageal reflux disease; HR, hazard ratio.

## 3 Results

### 3.1 Barrett's Esophagus

#### 3.1.1 Pathophysiology

Metaplasia occurs when one type of adult cell is replaced by another due to chronic tissue damage. In the case of GERD‐related esophageal injury, Barrett's metaplasia develops as mucus‐secreting columnar cells replace the damaged squamous esophageal epithelium. The exact origin of these metaplastic cells remains unclear. One hypothesis suggests that GERD alters the expression of key transcription factors, either triggering mature squamous cells to transform into columnar cells (trans‐differentiation) or directing immature progenitor cells toward columnar rather than squamous differentiation (trans‐commitment) [5]. Alternatively, progenitor cells, particularly bearing the Lgr5 stem cell marker from the gastric cardia, may migrate proximally. In support of this pathogenesis, there is a gradual transition from gastric‐type mucosa to intestinal metaplasia (IM) in cells from the proximal stomach into the esophagus, and there is a migratory front of metaplastic change rather than isolated metaplasia. As such, BE is generally not found emerging as islands within squamous epithelium (as would be expected from squamous cell transformation) but is rather a continuum of Barrett's epithelium marching from the gastric mucosa. Clonal analyses using mitochondrial DNA mutations (and other markers including LOH and p53) also support that BE arises from adjacent cardia and have a common clonal origin from progenitor cells there. Animal (rodent) models also support the migration model over trans differentiation of esophageal squamous cells [6]. Other potential sources of BE progenitor cells include submucosal glands of the esophagus, residual embryonic cells at the EGJ, and circulating bone marrow‐derived cells [7]. This has implications in the management of BE and its neoplastic progression since it suggests the future role of ablating the cardia, reducing glands buried below the neo‐squamous epithelium after ablation to reduce BE recurrence [8], and surveying for potential neoplasia at the cardia [9].

#### 3.1.2 Diagnosis

The diagnosis of BE presently requires endoscopic confirmation of columnar mucosa extending above the EGJ and lining the distal esophagus, along with biopsy evidence of IM. The gastroesophageal junction is identified endoscopically as the most proximal extent of the gastric folds. Characteristically, salmon‐colored columnar mucosa extends circumferentially or in tongue‐shaped projections above the gastroesophageal junction. Biopsies taken at this level confirm replacement of esophageal stratified squamous epithelium by IM containing goblet cells, which is essential for the diagnosis [5]. Chandrasoma et al. described these events in 2003 in endoscopic biopsies taken from 959 consecutive patients [10].

Screening for BE is recommended for individuals with multiple risk factors, including GERD, older age, white race, male sex, family history, obesity, alcohol use, and smoking. However, the relative importance of these factors remains unclear. Current screening strategies, which are based on endoscopy, have limitations, such as the lack of randomized trials proving their benefit, the high percentage of EAC cases without prior GERD symptoms, and the invasiveness and cost of endoscopy. To address these issues, risk‐based screening models that incorporate demographic and historical factors have been proposed to improve efficiency and cost‐effectiveness [11, 12].

**FIGURE 1:**
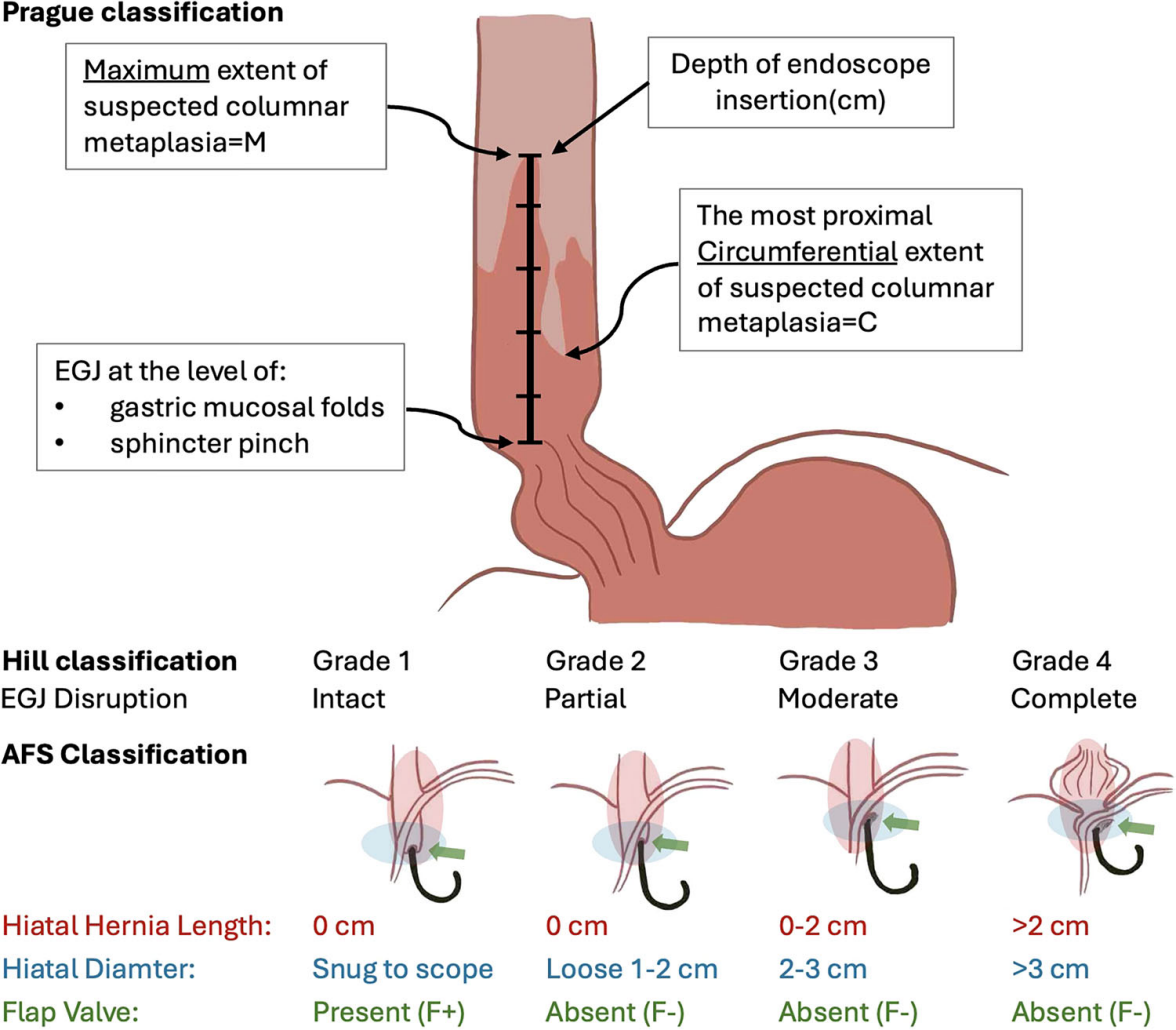
Key components of a standardized endoscopic report, including the Prague, Hill, and AFS classifications. Abbreviations: AFS, American Foregut Society; EGJ, esophagogastric junction.

Endoscopy is the gold standard for diagnosing BE. There are no clear recommendations on whether to perform screening endoscopy in patients without symptoms. Endoscopy is usually performed after the clinical diagnosis of reflux or dyspepsia, when symptoms persist despite treatment, or if alarm symptoms (e.g., dysphagia, weight loss, gastrointestinal (GI) bleeding) are present. The use of standardized criteria is crucial for the accurate endoscopic assessment of BE, supporting consistent risk stratification and clinical decision‐making [13]. According to the European Society of Gastrointestinal Endoscopy (ESGE) Guidelines [14], endoscopy reports should include: the position of the EGJ and diaphragmatic pinch; the extent of BE using the Prague classification (including proximal islands); in addition to lesion size (mm) and macroscopic appearance using the Paris classification; the presence or absence of erosive esophagitis using the Los Angeles classification. The Prague classification is a standardized system for describing BE based on the circumferential (C) and maximal (M) extent of visible columnar‐lined epithelium above the gastroesophageal junction (Figure 1) [15]. In addition to these classifications, the endoscopic assessment of the EGJ is essential for evaluating antireflux barrier integrity. The Hill classification grades the gastroesophageal flap valve from Grade I, a tightly closing valve, to Grade IV, a wide‐open junction with a large hiatal hernia; Grades I–II are generally considered normal, whereas Grades III–IV reflect significant EGJ dysfunction associated with reflux disease. Owing to its subjectivity and limited reproducibility, the American Foregut Society (AFS) has proposed a revised system that incorporates axial hernia length, hiatal diameter, and flap valve integrity, offering a more standardized and clinically relevant framework to guide therapeutic decision‐making [16].

Biopsies should be obtained according to the Seattle protocol (four‐quadrant biopsies at 1–2 cm intervals along the BE segment and targeted biopsies of any suspicious lesions) [17]. The diagnostic yield of BE in patients with symptomatic GERD or dysphagia is 3%–12%. A definitive diagnosis requires both endoscopic and histopathological confirmation of columnar‐lined epithelium. The ESGE defines BE as ≥1 cm of columnar epithelium visible endoscopically, due to high variability in diagnosing shorter segments and their low cancer risk [18, 19, 20].

Biopsies in BE have been shown to have low sensitivity, particularly in detecting early stage neoplasia, due to the patchy distribution of dysplastic lesions, with a significant portion remaining underdiagnosed [21]. A review found that 25% of EAC cases are missed within 1 year of negative endoscopy, highlighting the need for enhanced surveillance strategies, such as early repeat endoscopy and advanced imaging, to improve detection and reduce long‐term costs [22].

#### 3.1.3 Novel and Emerging Screening Techniques for BE

Many new strategies have been described that have the potential to revolutionize screening for BE. These include cell collection devices (Cytosponge, EsophaCap, EsoCheck), coupled with biomarker assays, and enhanced imaging. The Cytosponge is a swallowable capsule that collects esophageal cells for analysis, showing promise in large clinical trials [23].

Recent evidence supports the growing role of p53 immunohistochemistry (IHC) as an adjunct tool in the risk stratification and diagnosis of BE. Abnormal p53 expression—defined by either overexpression or complete loss of nuclear staining—has been shown to correlate strongly with *TP53* mutations and is associated with a significantly increased risk of progression to HGD or EAC, even in nondysplastic BE [24]. Importantly, p53 IHC improves diagnostic consistency, particularly in cases with indefinite for dysplasia, and has been shown to reduce interobserver variability among pathologists. In several studies, including large prospective cohorts, p53 IHC outperformed H&E staining alone in predicting disease progression [25]. Despite its promise, the interpretation of p53 staining patterns remains dependent on standardization, and further work is needed to harmonize cutoff values and integrate it into routine practice. Nevertheless, p53 IHC represents one of the most clinically validated biomarkers currently available for BE [26, 27].

Image‐based screening includes trans‐nasal unsedated endoscopy (TNE) and swallowable imaging capsules. Fast‐acquisition video capsules (PillCam ESO2) and tethered or magnet‐assisted capsule endoscopy (MACE, NaviCam) have been described, as well as Tethered Capsule Endomicroscopy, which uses optical coherence tomography (OCT). TNE employs thin‐caliber endoscopes to visualize the esophagus without the need for moderate sedation, but it requires practitioner training. Unlike confocal endomicroscopy, OCT allows real‐time broad‐field images of 3–10 micron axial resolution, but subsurface information of approximate 3 mm depth [28, 29]. These techniques offer cost‐effective, minimally invasive alternatives with high accuracy [30]. These methods could improve early BE detection and reduce reliance on endoscopy with biopsies [31].

Also on the horizon are breath tests for detecting volatile organic compounds (VOCs) and blood‐based tests [31]. The electronic nose (eNose) detects VOCs in breath and has demonstrated high sensitivity for BE detection [32].

Of these tools, the ESGE recommended that a swallowable nonendoscopic cell collection device, such as the Cytosponge, combined with a cytopathologic assessment and biomarker Trefoil‐factor 3, can be used as an alternative to endoscopy for case finding of BE. However, other nonendoscopic strategies could not be recommended yet [20].

Wide‐area transepithelial sampling with three‐dimensional analysis (WATS‐3D) uses a stiff endoscopic brush to sample a larger area of BE mucosa, reducing sampling errors and improving dysplasia detection. Analyzed by a neural network, WATS‐3D highlights areas with dysplasia for pathologists. When used in addition to traditional forceps biopsy (FB), WATS‐3D provides an incremental yield of dysplasia (7.2%) and HGD/EAC (2.1%), and it may identify progression missed by FB alone [33].

The TissueCypher Barrett's Esophagus Assay, validated in five multi‐institutional studies, predicts progression from nondysplastic, “indefinite,” LGD, HGD, and EAC, and detects HGD/EAC missed on endoscopy. This fluorescence imaging platform provides a risk score to classify patients’ likelihood of progressing to HGD/EAC within the ensuing 5 years. The test significantly impacts physician decision‐making, upstaging high‐risk scores and downstaging low‐risk scores, improving patient outcomes, and reducing unnecessary procedures [34]. The TissueCypher score, combined with genetic analysis, could provide individualized risk scores that help identify patients who are likely to benefit from intervention.

More research data are needed to effectively implement alternative screening methods into clinical practice. Additionally, proper training for personnel is essential to ensure their successful integration and use.

#### 3.1.4 Histological Diagnosis

The histologic diagnosis of BE remains debated, with the American College of Gastroenterology (ACG) requiring specialized IM for diagnosis, while the British Society of Gastroenterology and the International Benign Barrett's and Cancer Task Force allowing for columnar epithelium with or without IM. IM is linked to a higher risk of malignant progression, but studies show varying results regarding its significance in cancer risk. The ACG recommends obtaining at least eight biopsies to improve diagnostic yield and accuracy, but clinical adherence to this protocol is often suboptimal [35]. Despite differing diagnostic criteria, all guidelines emphasize the importance of segment length and IM presence in determining cancer risk, with surveillance strategies tailored to these factors [36]. The presence of circumferential Barrett's mucosa extending longitudinally for 3 cm or more is designated as long‐segment BE (LSBE), and the presence of circular Barrett mucosa less than 3 cm in length or the presence of noncircular Barrett's mucosa is designated as short‐segment BE (SSBE) [37]. LGD in BE is associated with a significant increased risk of progression to HGD and EAC. A meta‐analysis found that patients with LGD have an approximately fourfold higher risk of progression, compared to those with nondysplastic BE (OR: 4.25, 95% confidence interval [CI]: 2.58–7.00)​ [38]. Several studies have demonstrated that the risk of progression to HGD or EAC is significantly lower in patients with SSBE, as compared to those with LSBE. This was confirmed by a large multicenter study, which found that SSBE patients had a markedly reduced risk of developing HGD or cancer [39]. In summary, accurate staging and differentiation between LSBE, SSBE, LGD, and HGD are essential for risk stratification, surveillance planning, and treatment decisions to prevent the progression to EAC.

### 3.2 Surveillance, Therapy, and Prevention

#### 3.2.1 Surveillance

The development of EAC in BE is a gradual process, starting from metaplastic epithelium without dysplasia and then progressing to LGD, HGD, and eventually EAC, driven by esophageal acid and bile exposure as well as proteolytic enzymes such as pepsin. Therefore, endoscopic surveillance is essential for early detection of EAC in BE patients [40]. According to the ESGE guidelines, surveillance intervals for BE are based on dysplasia grade and BE segment length. For nondysplastic Barrett's esophagus (NDBE), the ESGE recommends surveillance endoscopy every 5 years for SSBE (<3 cm) and every 3 years for LSBE (≥3 cm), reflecting the increased risk of progression with longer segments [41, 42]. For LGD, the ESGE suggests more frequent surveillance, typically every 6−12 months, unless endoscopic eradication therapy is pursued, which is increasingly favored due to the higher risk of progression. For HGD, surveillance is not generally recommended as first‐line management; instead, endoscopic eradication therapy is preferred, but if surveillance is chosen, it should be performed every 3 months [43]. In cases with BE ≥10 cm, referral to a BE expert center is advised. Surveillance is not recommended for irregular Z‐lines or columnar epithelium <1 cm without IM. If dysplasia or EAC is detected, confirmation by a second experienced pathologist is required. In the presence of visible lesions, both targeted and random four‐quadrant biopsies should be performed, and management depends on endoscopic suspicion and histology. High‐quality surveillance includes counseling of patients, careful inspection of the entire metaplastic segment with both high‐definition white light and narrow band imaging or other digital chromoendoscopy modality, followed by appropriate tissue sampling for all mucosal abnormalities and systematic biopsies of the remaining visibly abnormal tissue. Advanced imaging methods may be used, but this approach lacks universal support. The cost‐effectiveness of routine surveillance is debated, with some advocating for high‐risk patients only. Scoring systems have been developed to identify these patients, but further studies are needed for validation [18, 44]. In summary, surveillance intervals are shorter for longer Barrett's segments and for higher grades of dysplasia, with the ESGE and other major societies recommending 3–5 years for NDBE (depending on segment length), 6–12 months for LGD, and 3 months for HGD if not treated endoscopically.

#### 3.2.2 Artificial Intelligence in Screening, Diagnosis, and Management of BE

Artificial intelligence (AI) can assist in managing BE using machine learning algorithms and deep learning techniques, which improve diagnostic accuracy. AI models are applied to endoscopic images to detect patterns particularly associated with dysplasia and early stage esophageal cancer. Similarly, AI has been applied to OCT imaging to detect neoplastic tissues [45]. These AI systems can help in risk stratification, predicting which patients are more likely to develop EAC, and guiding therapeutic decisions. Furthermore, AI‐driven tools provide continuous monitoring, improving surveillance and allowing for early intervention, which is crucial for preventing the progression of BE to EAC [46, 47, 48, 49].

#### 3.2.3 GERD Therapy

The ESGE suggests a proton pump inhibitor (PPI; standard dose‐omeprazole 40 mg or its dose equivalent‐once daily) for chemoprevention in patients with BE [20]. Chronic acid reflux in BE can cause inflammation, DNA damage, and increased cell proliferation, contributing to carcinogenesis, underscoring the importance of aggressive GERD treatment. Bile reflux, which can induce DNA damage and promote carcinogenesis, may not be prevented by PPI use, and its presence adds complexity to BE pathogenesis. The role of PPIs in chemoprevention, especially in asymptomatic or short‐segment BE cases, remains controversial [5].

While PPIs effectively manage reflux esophagitis, up to 40% of patients still experience persistent GERD symptoms, and PPIs show limited efficacy in extra‐esophageal GERD manifestations, like laryngitis and chronic cough. Retrospective studies suggest PPIs may reduce BE progression to cancer, but this remains unproven [50].

Additionally, studies in both humans and rats demonstrate that reflux involves more than just acid. Gastric and duodenal juices cause inflammation, cell proliferation, and mutagenic effects through bile acids, which may exacerbate BE and EAC development. Thus, long‐term PPI therapy, especially in BE patients with bile reflux, should be approached cautiously [51].

In an in vitro study, Sun et al. investigated how bile acids contribute to the development of BE, focusing on their inflammatory and proliferative effects. They found that while acid reflux alone did not induce BE, bile acids played a key role by promoting cell proliferation and inflammation in esophageal cells [52]. Ursodeoxycholic acid (UDCA), the most hydrophilic of the bile acids, was shown to protect against bile acid and low pH‐induced oxidative stress and oxidative DNA damage and to also modulate the expression of enzymes associated with protection against oxidative stress in cultured esophageal cells [53]. As such, UDCA could theoretically help mitigate the harmful effects of bile acids in BE. In a study by Banerjee et al., high‐dose UDCA supplementation in patients with BE increased cytoprotective bile acids and decreased cytotoxic bile acids in the gastric fluid. However, no significant changes were observed in oxidative DNA damage, cell proliferation, or apoptosis in the BE epithelium. Further research on UDCA's impact on genomic alterations and its potential role with PPI therapy in preventing neoplastic progression is needed [54].

Furthermore, potassium‐competitive acid blockers (PCABs) have shown promise in RCTs, as summarized in a meta‐analysis by Simadibrata et al. These authors showed a superior outcome compared to PPI in first‐line *Helicobacter pylori* eradication and erosive esophagitis. Future research will be needed to evaluate the impact of PCABs on EAC development in patients with BE [55].

#### 3.2.4 Endoscopic Ablation

The ESGE Guidelines recommend endoscopic therapy for BE patients with HGD or early stage EAC, using radiofrequency ablation, endoscopic mucosal resection, or cryotherapy, aiming at the eradication of both the dysplasia and IM. For LGD, treatment may be considered, and it is based on risk factors, with surveillance typically recommended. Radiofrequency ablation is preferred for dysplasia due to its high efficacy, while endoscopic mucosal resection is used for visible (nodular) lesions or early EAC. Post‐treatment surveillance is essential to monitor recurrence [20].

#### 3.2.5 Antireflux Surgery for Preventing Progression

Antireflux surgery (ARS) restores the frequently defective antireflux barrier in BE by reducing acid and/or duodenal reflux. It is primarily indicated for patients unwilling to take PPIs long‐term, those with refractory symptoms [56], or for patients with persistent esophagitis unresponsive to medical therapy [57]. Surgical therapy has a theoretical advantage in that it can control reflux of all types of gastric contents into the esophagus [58]. Patients with objectively confirmed GERD who have typical symptoms (heartburn, regurgitation), a positive pH test, and a good response to PPI therapy are most likely to benefit from surgical intervention. Conversely, those with atypical or extraesophageal symptoms, or poor response to PPIs, require a rigorous diagnostic workup to confirm reflux as the etiology before surgery is considered, as outcomes are otherwise suboptimal [59]. Preoperative evaluation should include upper endoscopy, esophageal manometry, and ambulatory pH monitoring to exclude alternative diagnoses (e.g., achalasia, functional heartburn) and to assess esophageal motility. Manometry is essential for identifying patients with impaired peristalsis, who are better suited to partial fundoplication (e.g., Toupet or Dor) rather than total fundoplication, due to lower risk of postoperative dysphagia [60]. For patients with large hiatal hernias, extensive mediastinal dissection and crural repair are required to ensure durable results [59].

**FIGURE 2:**
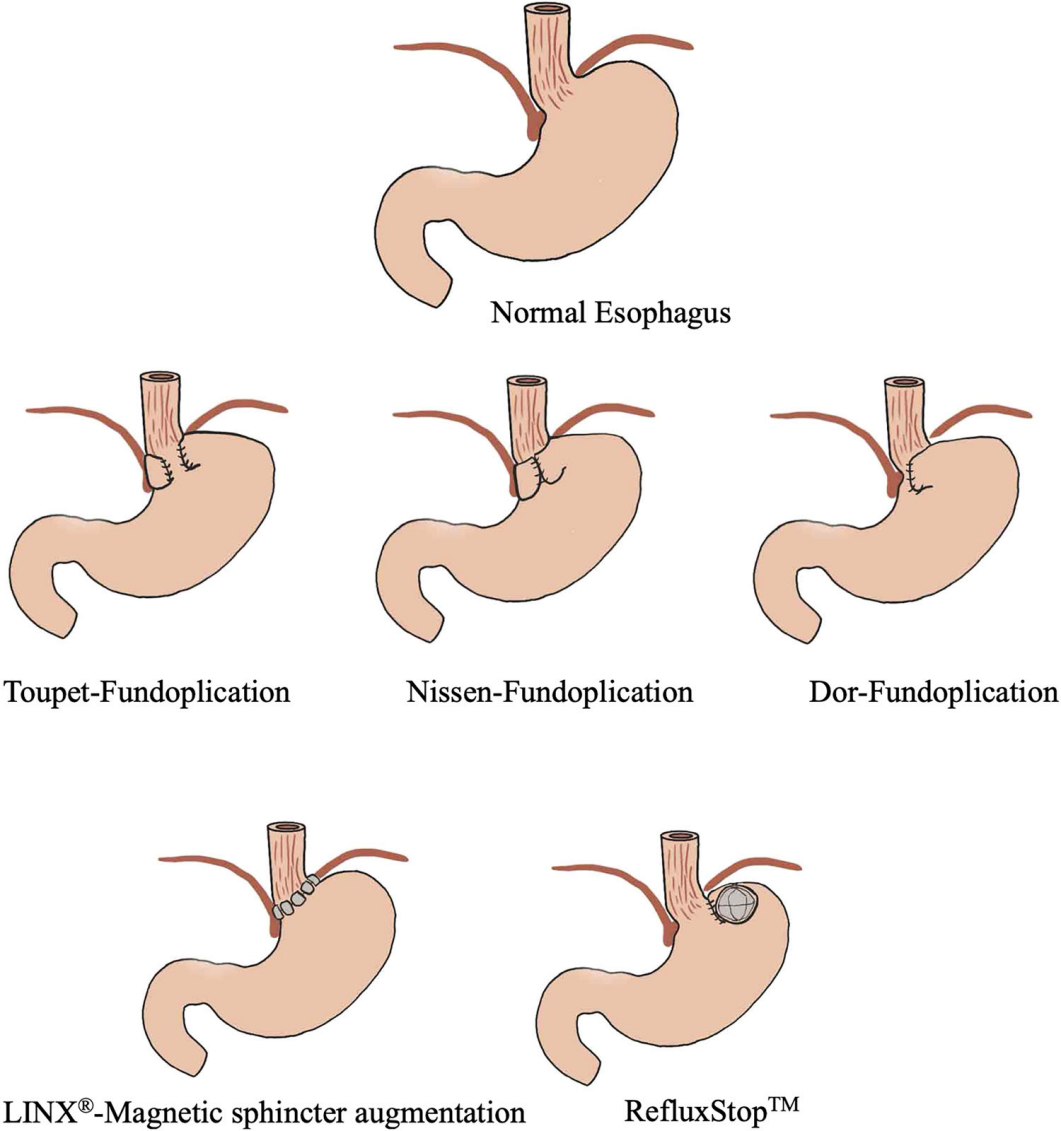
The most common types of reconstruction used in antireflux surgery (ARS), including partial fundoplications (Toupet and Dor), Nissen fundoplication, and the implantation of antireflux devices such as magnetic sphincter augmentation or the RefluxStop system.

The most commonly performed surgical procedure for GERD is laparoscopic fundoplication associated with cardioplasty, which enhances the EGJ's ability to prevent reflux into the esophagus [61]. The most common types of reconstruction are summarized in Figure 2. Potential long‐term complications include dysphagia, gas bloat syndrome, and a higher risk of esophageal dysmotility. For these reasons, a thorough preoperative assessment and post‐surgical monitoring are necessary to achieve optimal outcomes [62]. Since many patients with BE exhibit esophageal dysmotility, preoperative functional diagnostics are crucial to appropriately adapt the surgical technique for antireflux procedures [63]. New technologies in ARS (i.e., LINX) have been developed to minimize potential side effects of conventional ARS. Currently, the impact of these technologies on the regression of BE is uncertain [64]. Laparoscopic reconstruction of the angle of His is a safe and effective alternative to traditional fundoplication for GERD and hiatal hernia. This technique provides comparable antireflux efficacy while reducing operative time, blood loss, hospital stay, and postoperative complications, making it a less invasive and better‐tolerated option. Magnetic sphincter augmentation (MSA) or LINX is a significant advancement, restoring the antireflux barrier with minimal anatomical disruption. Long‐term follow‐up revealed no new safety concerns, and the procedure demonstrated durable benefits, including reduced acid exposure, symptom improvement, higher patient satisfaction, and elimination of PPI use [65]. As a first‐line surgical option, MSA involves minimal dissection and preserves gastric anatomy, highlighting its safety and efficacy for GERD treatment [66]. The RefluxStop device, introduced in 2018, treats GERD by restoring the antireflux barrier without esophageal encirclement. Three‐year results confirm its safety, effectiveness, and long‐term success, with no device‐related complications and significant improvements in GERD symptoms (93% GERD‐HRQL score) and complete cessation of PPI use [67].

**FIGURE 3:**
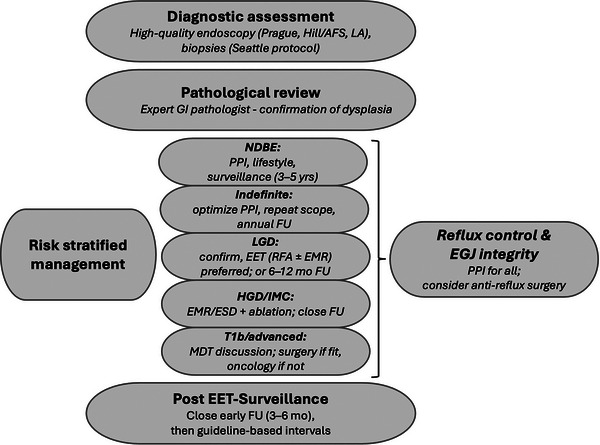
Summary of the diagnostic and therapeutic management algorithms for Barrett's esophagus (BE). Abbreviations: AFS, American Foregut Society; EET, endoscopic eradication therapy; EGJ, esophagogastric junction; EMR, endoscopic mucosal resection; ESD, endoscopic submucosal dissection; FU, follow‐up; GI, gastrointestinal; HGD, high‐grade dysplasia; IMC, intramucosal carcinoma; LGD, low‐grade dysplasia; NDBE, nondysplastic Barrett's esophagus; PPI, proton pump inhibitor; RFA, radiofrequency ablation.

Ultimately, the choice of procedure should be individualized, taking into account patient anatomy, motility, comorbidities, and preferences, and performed in centers with comprehensive diagnostic capabilities and surgical expertise [68]. A summary on the diagnosis and treatment of BE is shown in Figure 3.

### 3.3 Effect of Surgery on BE Development and Progression

Several studies addressing the development of BE after ARS have been performed, and are discussed below and summarized in Table 1. Parrilla et al. conducted a randomized prospective study comparing medical and surgical treatments for BE. A total of 101 patients were assigned to either medical therapy (*n* = 43) or ARS (*n* = 58). After a median follow‐up of 5 years for the medical group and 6 years for the surgical group, satisfactory clinical outcomes (rated as excellent to good) were observed in 91% of patients in both groups. However, persistent inflammatory lesions were significantly more common in the medical treatment group. The length of the metaplastic segment remained unchanged in all patients. Postoperative functional assessments indicated a significant reduction in the median percentage of total time with esophageal pH<4. Despite this, 15% of surgical patients continued to exhibit pathological acid reflux. HGD developed in 5% of the medical group and 3% of the surgical group (NS), with no cases of malignancy reported following successful ARS [69].

Similarly, a systematic review by Chang et al. analyzed the impact of ARS versus medical therapy on EAC incidence in BE patients. The study followed 700 surgically treated patients for a total of 2939 patient‐years and 996 medically treated patients for 3711 patient‐years. The incidence of EAC was 2.8 per 1000 patient‐years (95% CI, 1.2–5.3) in the surgical group and 6.3 per 1000 patient‐years (95% CI, 3.6–10.1) in the medical group (*p* = 0.034). Controlled studies within the review reported incidence rates of 4.8 per 1000 patient‐years (95% CI, 1.7–11.1) for surgical patients and 6.5 per 1000 patient‐years (95% CI, 2.6–13.8) for medical patients (*p* = 0.320). The probability of disease progression was 2.9% (95% CI, 1.2–5.5) in the surgical group and 6.8% (95% CI, 2.6–12.1) in the medical group (*p* = 0.054). Disease regression was observed in 15.4% (95% CI, 6.1–31.4) of surgical patients, compared to 1.9% (95% CI, 0.5–5.9) in the medical group (*p* = 0.004) [70].

Maret‐Ouda et al. conducted a systematic review and meta‐analysis assessing the impact of ARS on the incidence of EAC in GERD patients. Data from 10 studies were analyzed, revealing a pooled incidence rate ratio of 0.76 (95% CI, 0.42–1.39) for EAC in surgically treated patients, as compared to those receiving medical therapy. When focusing specifically on BE patients, the pooled incidence rate ratio was 0.46 (95% CI, 0.20–1.08). Compared to the general population, the incidence rate ratio for EAC in post‐ARS patients was 1.34 (95% CI, 0.73–2.47). These findings suggest a potential reduction in EAC risk following ARS, particularly among patients with BE, although the results did not reach statistical significance [71].

In a national population‐based cohort study in England, Markar et al. analyzed 838,755 GERD patients (22,231 with ARS) and 28,372 BE (737 with surgery) to assess esophageal cancer risk. Over the study period (2000–2012), ARS in GERD patients was associated with a reduced cancer risk (hazard ratio [HR]: 0.64, 95% CI: 0.52–0.78). However, in BE patients, the risk reduction was not statistically significant (HR: 0.47, 95% CI: 0.12–1.90). Sensitivity analysis found no cases of EAC in surgically treated GERD patients, while Barrett's patients had an HR of 0.90 (95% CI: 0.25–3.21). These findings suggest a potential protective effect of ARS in GERD, but remain inconclusive for BE [72].

Later, in 2021, Maret‐Ouda et al. demonstrated that EAC risk remained stable over time in both surgical and nonsurgical treatment groups, suggesting that neither approach effectively reduces long‐term cancer risk in GERD patients. A comprehensive cohort study conducted across five Nordic countries evaluated the long‐term risk of EAC following ARS. The study included 48,414 patients who underwent ARS between 1965 and 2014, contributing a total of 477,827 person‐years of follow‐up. During this period, 177 cases of EAC were identified. The standardized incidence ratio (SIR) for EAC in this cohort was 2.3 (95% CI, 2.0–2.7), indicating a more than twofold increased risk, as compared to the general population. Notably, the elevated risk persisted beyond 15 years post‐surgery, with an SIR of 2.5 (95% CI, 1.8–3.3). These findings suggest that while ARS effectively treats GERD symptoms, it does not eliminate the long‐term risk of developing EAC [73].

In the most recent multinational, population‐based cohort study from 2024, Åkerström et al. analyzed 33,939 BE patients, comparing EAC incidence between those who underwent ARS (*n* = 542) and those receiving antireflux medication (*n* = 33,397). Over a follow‐up period of up to 32 years, 14 cases of EAC (2.6%) were identified in the surgical group, compared to 437 cases (1.3%) in the medication group. The adjusted HR for developing EAC in the surgical group was 1.9 (95% CI, 1.1–3.5) compared to the medication group. Notably, the risk appeared to increase over time, with an HR of 1.8 (95% CI, 0.6–5.0) within 1–4 years post‐surgery, rising to 4.4 (95% CI, 1.4–13.5) after 10–32 years. These findings indicate that ARS does not reduce EAC risk compared to medical therapy in BE patients [74].

### 3.4 Obesity and BE

Obesity, especially central adiposity, is an independent risk factor for BE, even after adjusting for GERD symptoms [75]. Visceral fat contributes to increased intra‐abdominal pressure and promotes reflux, but also induces local inflammation via adipokines such as leptin and adiponectin [76, 77]. Roux‐en‐Y gastric bypass (RYGB) is associated with significant weight loss and marked improvement of reflux symptoms. Unlike sleeve gastrectomy, RYGB reduces both acid and bile exposure to the esophagus [78]. Several retrospective studies have reported regression or stabilization of BE after RYGB [79, 80]. However, evidence is limited and mostly observational; its long‐term cancer‐preventive effects remain unclear [81]. Although RYGB is often preferred in patients with obesity with GERD or BE, it is not currently recommended solely for BE management without obesity‐related indications.

## 4 Discussion

Studies on the preventive effect of ARS in BE have yielded varying results [69, 70, 71, 72, 73, 74]. One possible explanation is that patients undergoing surgery often present with an advanced stage of the disease, as they are typically referred only after medical therapy has failed. As a result, many have a long history of GERD symptoms, large hiatal hernias, severe esophagitis, and long segments of columnar‐lined esophagus with or without dysplastic BE [64]. Additionally, they often exhibit a lower esophageal sphincter tone, impaired esophageal motility, and abnormal esophageal pH monitoring. Since these are well‐established risk factors for malignant progression, it is important to consider that patients selected for surgery may already have a predisposition to an increased cancer risk, which could influence study outcomes [64, 71, 82].

Lee et al. reviewed the efficacy, cost‐effectiveness, and patient selection for ARS, highlighting that, while ARS is effective, it is underutilized. Proper patient selection, requiring endoscopy or esophageal pH monitoring, is essential, and studies show that ARS costs are comparable to long‐term PPI use after 9 years [82]. In a retrospective study, Salvador et al. demonstrated that laparoscopic ARS is effective and durable (for >20 years) in patients with uncomplicated GERD and, to a lesser extent, in those with a large hiatal hernia [83]. Long‐term PPI use has been linked to several potential adverse effects, including chronic kidney disease, increased risk of *Clostridium difficile* infection, osteoporosis, and gastric cancer. Additionally, discontinuing PPIs may cause rebound acid hypersecretion, which can be mitigated by gradual dose reduction. Most of the evidence comes from observational studies, and the long‐term consequences remain uncertain [61].

Markar et al. demonstrated that patients with GERD who underwent ARS had a reduced risk of esophageal cancer compared to nonoperated GERD patients. Although limited by low statistical power, the study also suggested a decreased risk of esophageal cancer when comparing ARS with PPI therapy. Similar indications were found for patients with BE, suggesting that surgical intervention may also reduce the risk of malignant transformation in this patient group. Although published in 2003, the study by Oelschlager et al. remains a key piece of evidence supporting ARS in BE. They showed significant symptom improvement, a restored barrier function, and regression of BE in over 55% of patients with SSBE. These findings support early surgical intervention, especially in SSBE, while the benefit in LSBE remains less clear [84]. These findings highlight the importance of early detection and treatment of GERD, as well as the potential role of ARS in the prevention of BE and EAC. It is important to note that ARS is typically considered a good option for patients with GERD when quality of life is significantly affected, and long‐term PPI use is necessary. In contrast, BE alone does not constitute an indication for surgery, unless there are additional risk factors, such as progression to dysplasia or carcinoma [72].

## 5 Conclusion

In conclusion, the adoption of patient‐tailored interventions, combined with the integration of AI, holds significant promise in preventing esophageal tissue damage and, consequently, the progression of BE to EAC. While these personalized approaches have shown varying degrees of success, further research is essential to evaluate their long‐term efficacy and clinical outcomes. Furthermore, it is important to acknowledge that even with successful ARS, patients need to be included in surveillance programs in order to rule out progression.

Given the current variability in outcomes and methodologies, future studies should prioritize large‐scale, well‐designed comparative trials with homogeneous patient populations. This approach will facilitate the development of evidence‐based, optimized treatment protocols for EAC prevention in individuals with BE.

## Author Contributions

D.K.: Design of the review, writing the review, designing the figures, correction; C.E.: Writing the review, designing the figures; R.A.: Contributed to the preparation of the manuscript, review; G.J.: Contributed to the preparation of the manuscript, review; G.T.: Contributed to the preparation of the manuscript, review; K.J.V.: Contributed to the preparation of the manuscript, review, B.Z.: Contributed to the preparation of the manuscript, review; M.J.Z.: Contributed to the preparation of the manuscript, review; Q.H.: Contributed to the preparation of the manuscript, review; H.M.: Contributed to the preparation of the manuscript, review; E.J.S.: Contributed to the preparation of the manuscript, review; J.O.C.: Contributed to the preparation of the manuscript, review; S.F.S.: Contributed to the preparation of the manuscript, review.

## Conflicts of Interest

The authors do not have any competing interests associated with the preparation of the manuscript.
